# Moving house: long-term dynamics of corticosterone secretion are unaltered in translocated populations of a rare reptile (the tuatara, *Sphenodon punctatus*)

**DOI:** 10.1093/conphys/cov014

**Published:** 2015-04-11

**Authors:** Lindsay E. Anderson, Alison Cree, David R. Towns, Nicola J. Nelson

**Affiliations:** 1 Allan Wilson Centre for Molecular Ecology and Evolution, School of Biological Sciences, Victoria University of Wellington, PO Box 600, Wellington 6140, New Zealand; 2 Department of Zoology, University of Otago, PO Box 56, Dunedin 9054, New Zealand; 3 Ecosystem Development Team, Science and Technical Group, Department of Conservation, Private Bag 68-908 Newton, Auckland 1145, New Zealand

**Keywords:** Corticosterone, reptile, stress, translocation, tuatara

## Abstract

Translocations are an important conservation tool used to restore at-risk species to their historical range. Unavoidable procedures during translocations, such as habitat disturbance, capture, handling, processing, captivity, transport and release to a novel environment, have the potential to be stressful for most species. In this study, we examined acute and chronic stress (through the measurement of the glucocorticoid corticosterone) in a rare reptile (the tuatara, *Sphenodon punctatus*). We found that: (i) the acute corticosterone response remains elevated during the initial translocation process but is not amplified by cumulative stressors; and (ii) the long-term dynamics of corticosterone secretion are similar in translocated and source populations. Taken together, our results show that translocated tuatara are generally resistant to cumulative acute stressors and show no hormonal sign of chronic stress. Translocation efforts in tuatara afford the potential to reduce extinction risk and restore natural ecosystems.

## Abstract (precis)

Translocations are an important conservation tool; however, the process is a potential stressor for most species. We evaluated effects of translocation on plasma corticosterone in a rare reptile (the tuatara). We found that translocated tuatara are resistant to cumulative stressors and show no hormonal sign of chronic stress.

## Introduction

Translocations are human-assisted movements of living organisms from one area to another and are an important tool for conservation efforts and population restoration of species at risk (Armstrong and Seddon, 2008; Ewen *et al*., 2012; Seddon *et al*., 2014). The International Union for Conservation of Nature (IUCN) recognizes two types of conservation translocation to restore populations, namely (i) reinforcements, in which individuals are released into an existing population of conspecifics to enhance the sustainability of populations, and (ii) reintroductions, in which individuals are released in a historically occupied area in order to re-establish a population after extirpation. Although these types of movements are ultimately aimed at helping species, the translocation process is inherently stressful, because associated procedures, such as habitat disturbance, capture, handling, processing, captivity, transport and release to a novel environment, are necessary and unavoidable (Germano and Bishop, 2009; Dickens *et al*., 2010; Parker *et al*., 2012). In a recent review, Tarszisz *et al*. (2014) identified physiology as a key disciplinary area that is lacking attention in conservation translocations and also highlighted how physiological data can improve short- and long-term translocation success.

In vertebrates, the acute stress response produces a rapid increase in glucocorticoid hormone secretion [corticosterone (CORT) or cortisol] to help individuals cope with immediate stressors (Wingfield *et al*., 1998); consequently, non-essential processes (such as reproduction and growth) are suspended until homeostasis returns. Although the stress response serves to promote immediate survival, prolonged or sustained CORT secretion (typically expected during translocations) can manifest as ‘chronic stress’ and is generally considered detrimental to overall health and fitness (i.e. the CORT-Fitness Hypothesis; Sapolsky *et al*., 2000; Wingfield and Sapolsky, 2003; Bonier *et al*., 2009; Parker *et al*., 2012; Almasi *et al*., 2013). In a recent review of the associations between stress and movement of animals, Teixeira *et al*. (2007) concluded that stress is a contributing factor to the success or failure of a translocation project. Stress induced by the initial translocation process and relocation to a novel environment increases the vulnerability of individuals to reproductive failure, disease, starvation, predation and long-range dispersal, thereby decreasing the chance that individuals will survive and that a self-sustaining population will result (Teixeira *et al*., 2007; Dickens *et al*., 2009, 2010; Parker *et al*., 2012).

Measuring and monitoring CORT secretion is the most widely used method for assessing stress in vertebrates (Wikelski and Cooke, 2006; Dickens *et al*., 2010; Sheriff *et al*., 2011). Although several factors relevant to translocation efforts influence CORT secretion, studies that assess and monitor stress (by way of CORT secretion) throughout and after the translocation process are limited (Germano and Bishop, 2009; Harrington *et al*., 2013; Tarszisz *et al*., 2014). Numerous studies have shown that associated procedures commonly applied in translocation programmes (such as capture, handling and transport) stimulate a significant stress response and influence CORT secretion (Fazio and Ferlazzo, 2003; Langkilde and Shine, 2006; Narayan and Hero, 2011; Bliley and Woodley, 2012; Baker *et al*., 2013; Fazio *et al*., 2014). Likewise, altered CORT secretion has been associated with variation of environmental factors, such as exposure to humans (French *et al*., 2010; Taylor *et al*., 2014) and novel predators (Berger *et al*., 2007; Rödl *et al*., 2007), change in food availability (Woodley *et al*., 2003; Kitaysky *et al*., 2007; Bryan *et al*., 2014), latitudinal differences (Silverin *et al*., 1997; Dahl *et al*., 2012; Eikenaar *et al*., 2012; Quirici *et al*., 2014) and habitat type (French *et al*., 2008; Zhang *et al*., 2011; Li *et al*., 2012; Bauer *et al*., 2013). In addition to experiencing acute stressors during the initial translocation process, translocated individuals released into new environments are faced with several survival challenges (such as finding food and shelter and avoiding predators); therefore, physiological stress is inevitable (Teixeira *et al*., 2007).

Here, we examine the acute CORT response and long-term dynamics of CORT secretion through the translocation process in a rare reptile, the tuatara (*Sphenodon punctatus*). The tuatara is a protected reptile endemic to New Zealand and is the only living representative of the reptilian order Rhynchocephalia (Jones and Cree, 2012). Although the tuatara is now considered non-threatened but ‘at-risk – relict’ (Hitchmough *et al*., 2013), translocations contribute to conservation and ecological restoration efforts and serve to re-­establish tuatara within their pre-human range (Hitchmough *et al*., 2013; Cree, 2014). In addition to easing extinction pressure, translocations also offer a chance to examine and address relevant research questions (Germano and Bishop, 2009; Miller *et al*., 2012; Cree, 2014). In 2012, wild tuatara were translocated to six island and mainland sanctuaries from two source populations (Lady Alice Island and Stephens Island/Takapourewa, New Zealand; Cree, 2014), which presented an excellent opportunity to examine the CORT response to translocation in multiple populations. Previous studies have examined patterns of CORT secretion in tuatara; in general, baseline CORT in tuatara is fairly low (with plasma concentrations typically 2–5 ng/ml), a significant CORT response to capture restraint is observed, and female reproductive condition, body temperature and season (but not time of day) are influential factors (Tyrrell and Cree, 1998; Tyrrell *et al*., 2000; Anderson *et al*., 2014). In a recent study examining CORT secretion in four populations of tuatara, we found that baseline CORT was similar among all populations; however, the CORT response varied with latitude, seabird density, sex ratio and genetic diversity (L. Anderson, N. Nelson, D. Towns and A. Cree, unpublished data). Although translocations of tuatara continue to take place, CORT secretion (as an indicator of stress) during and after the translocation process has not been examined. Comparing CORT secretion simultaneously in translocated and source populations of tuatara would allow detection of altered CORT secretion that is correlated with environmental and/or habitat change and that would be an indication of chronic stress.

This study had two aims. First, we examined the acute CORT response in tuatara at different stages of the initial translocation process and tested the prediction that the acute CORT response would be amplified with cumulative stressors. Second, we tested whether long-term changes in CORT secretion provide evidence of ‘chronic stress’ in three translocated populations (compared with the corresponding source populations as control animals). We made the following predictions: (i) baseline CORT (post-translocation) would be similar among all populations; and (ii) the CORT response (post-translocation) would be amplified in translocated populations that experienced a marked environmental and/or habitat change (e.g. a greater latitudinal shift). Moreover, body condition indices (mass relative to snout–vent length) in translocated populations (along with source populations as control animals) were examined, because chronic stress can influence energy expenditure (Romero, 2002).

## Materials and methods

### Study design

**Figure 1:**
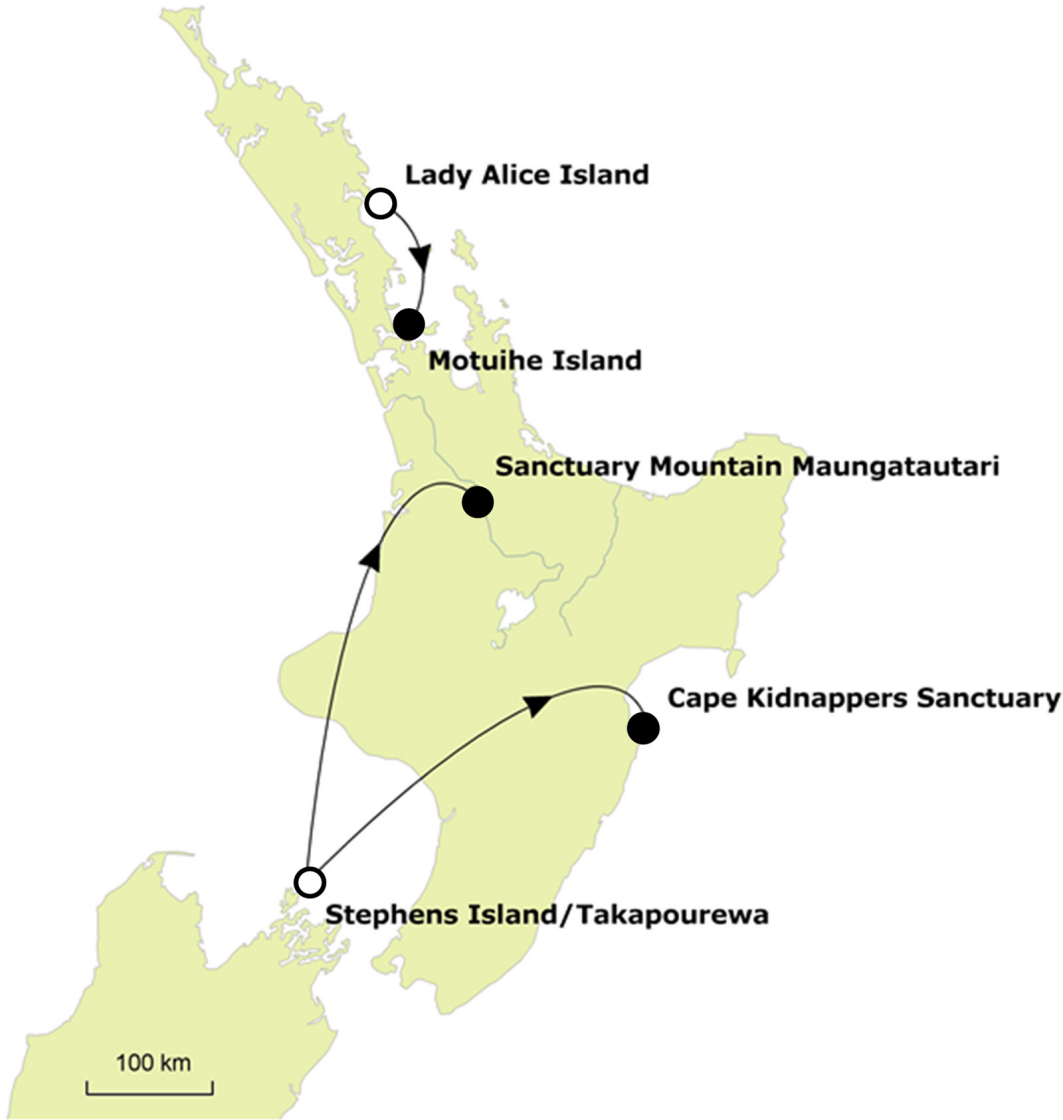
Populations involved in short- and long-term monitoring of physiological data (corticosterone) throughout a conservation translocation programme in New Zealand. Source populations (open circles) and translocated populations (filled circles) are shown.

**Figure 2:**
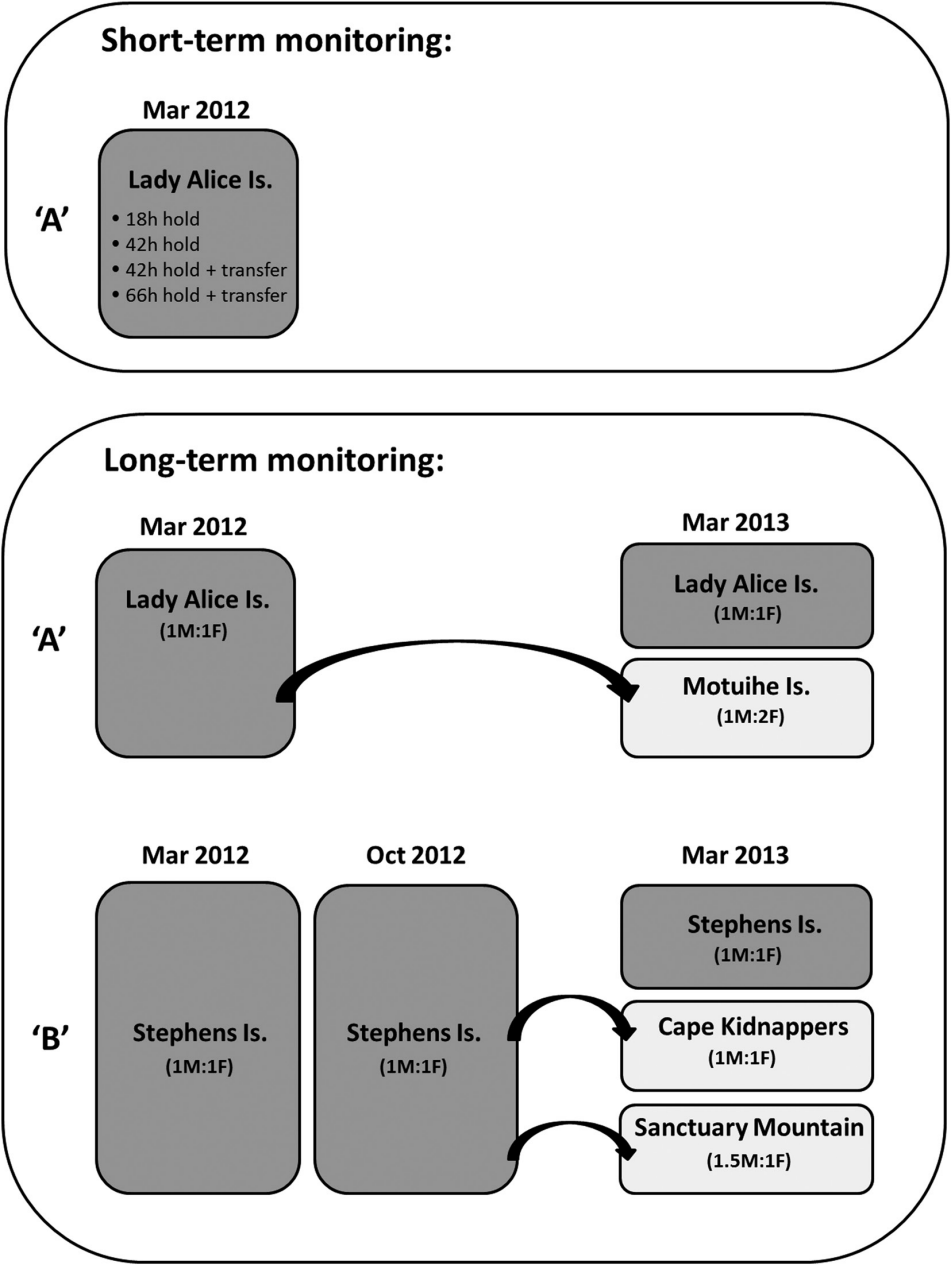
Schematic diagram of short-term (upper panel) and long-term monitoring (lower panel) during the translocations to Motuihe Island from Lady Alice Island in March 2012 (translocation ‘A’) and to Cape Kidnappers and Sanctuary Mountain Maungatautari from Stephens Island in October 2012 (translocation ‘B’). Short-term monitoring was carried out during translocation A only. Comparisons for long-term monitoring were made between source (dark grey) and translocated populations (light grey). The sex ratio (M:F) for each population is shown.

We took advantage of two planned translocations in New Zealand (Fig. 1) to examine short-term and long-term dynamics of CORT secretion in tuatara throughout the translocation process (Fig. 2). In translocation ‘A’ (March 2012), wild adult tuatara were translocated to Motuihe Island (35° 58′ S, 174° 43′ E; *n* = 60) from Lady Alice Island (35° 53′ S, 174° 43′ E). Lady Alice and Motuihe Islands are located in the Northern New Zealand regional climate zone, experiencing sub-tropical warm humid summers and mild winters (NIWA, 2014). In ­translocation ‘B’ (October 2012), wild adult tuatara were translocated to five locations from Stephens Island (40° 40′ S, 174° 00′ E), including Cape Kidnappers Sanctuary (39° 64′ S, 177° 09′ E; *n* = 40) and Sanctuary Mountain Maungatautari (38° 30′ S, 175° 33′ E; *n* = 50), which were the two locations monitored in this study. Stephens Island is located in the Northern South Island regional climate zone, and Cape Kidnappers Sanctuary is located in the Eastern North Island regional climate zone, both experiencing warm dry summers and mild winters (with frost). Sanctuary Mountain Maungatautari is located in the Central North Island regional climate zone, experiencing warm dry summers and cool winters (with frost and fog; NIWA, 2014). All translocation release sites offered suitable physical habitat for ­tuatara (with artificial burrows also provided) and social aspects, such as male:female sex ratio (Fig. 2), and tuatara densities were within the normal range.

#### Short-term monitoring: the acute corticosterone response at different stages of the initial translocation process

In translocation A only, we examined the acute CORT response (i.e. CORT secretion above baseline) through all stages of the initial translocation process, during which standard translocation protocols were followed (Towns *et al*., 1990; Cromarty and Alderson, 2013). In summary, adult tuatara (snout–vent length ≥170 mm) that had emerged from their underground burrows were captured by hand (between 20.00 and 04.00 h), and sex was identified by examining secondary sex characteristics, such as head size/shape, body shape, spine shape and crest development (Cree, 2014).

All individuals in the translocation programme were subjected to a capture-restraint ‘hold’ [which involved capture of individuals and initial holding (between 40 and 60 h) in cloth capture bags], processing (which involved handling, weighing, measuring and implantation of a passive integrated transponder tag) and transfer to the release site [which involved holding (between 6 and 10 h) in perforated cardboard postal tubes (10 cm × 50 cm), movement by foot to the helicopter pick-up site, a 30 min helicopter flight, unloading and a 30 min ­handing-over ceremony upon arrival at Motuihe Island].

To determine the acute CORT response at different stages of the translocation programme, we collected baseline CORT samples (following the blood sampling protocol described below in the ‘*Sampling protocol*’ section) from tuatara at capture (0 h; *n* = 54) and collected a second sample after one of the following: (i) an 18 h hold (*n* = 15); (ii) a 42 h hold (*n* = 14); (iii) a 42 h hold + process + transfer (*n* = 11); or (iv) a 66 h hold + process + transfer (*n* = 14). Tuatara do not show significant daily variation in baseline CORT (Tyrrell and Cree, 1998); therefore, the time of day at sampling is unlikely to contribute to variation in CORT secretion in this study.

#### Long-term monitoring: dynamics of corticosterone secretion post-translocation

Figure 2 presents a schematic diagram of samples obtained during translocations A and B and displays sex ratios (M:F) for each population. For all samples, adult tuatara (both sexes) were captured at night by hand (between 20.00 and 04.00 h). Upon capture, a baseline CORT sample was obtained, and after 3 h capture restraint in a cloth bag, a second sample was obtained to determine the CORT response. In translocation A, we collected samples from the source population (Lady Alice Island; LA) before translocation (March 2012) and from the source (LA) and translocated (Motuihe Island; Mot) populations at 12 months post-translocation (March 2013). In translocation B, we collected samples from the source population (Stephens Island; ST) before translocation (October 2012) and from the source (ST) and translocated [Cape Kidnappers (CK) or Sanctuary Mountain Maungatautari (MT)] populations at 6 months post-translocation (March 2013). Given that significant seasonal variation in CORT secretion has been observed between the breeding (March) and non-breeding (October) seasons in tuatara (L. Anderson, N. Nelson, D. Towns and A. Cree, unpublished data), we also analysed samples from the ST source population (obtained in a previous study; March 2012) for annual comparison. In order to determine whether the release site (within a translocated population) had a significant effect on CORT secretion, we collected post-­translocation samples from two separate release-site locations on Mot (site 1 = Orchards Bush and site 2 = Von Luckner's Bush) and at MT (site 1 = Tuatarium and site 2 = Northern Enclosure).

### Sampling protocol

In order to determine baseline CORT concentrations, a blood sample (up to 1 ml) was collected within 10 min of capture from the base of the tail with a heparinized 23-gauge needle and 1 ml syringe. After baseline samples were taken, individuals underwent capture restraint in a cloth capture bag and/or postal tube (3–66 h, depending on study), whereupon a second blood sample (up to 1 ml) was taken to determine the CORT response. Internal body temperature (*T*_b_) was recorded with a cloacal thermocouple (Fluke^®^ Multimeter, model 179; specified accuracy ±0.1°C; Fluke Corporation, Everett, WA, USA) prior to taking blood samples from each individual (both baseline and CORT response). After CORT response samples were obtained, individual mass (in grams) was determined (to the nearest ±5 g) with a 1000 g spring scale (Pesola AG, Baar, Switzerland), and snout–vent length (in millimetres), tail length (in millimetres) and tail regeneration length (in millimetres) were measured with a ruler. Body condition scores were generated for each individual as standardized residuals from a regression of log tail-corrected mass (Newman *et al*., 1994) and log snout–vent length (Schulte-Hostedde *et al*., 2005). Body condition scores were generated separately for each source population (ST and LA) and sex.

Depending on field conditions (i.e. electricity available or not), blood samples were separated either by centrifuge (5 min at 480 *g*) or under normal gravity for 6–8 h at 4°C (Reimers *et al*., 1983; Sheriff *et al*., 2011; Anderson *et al*., 2014). Plasma was transferred into cryogenic vials with a micropipette, stored in a cryogenic dry shipper (Thermo ScientificTM, Arctic ExpressTM Dual 10) or in a freezer at −20°C until return to the laboratory, and then stored at −80°C until assayed. Corticosterone was analysed with commercial enzyme immunoassay kits (Cayman Chemical Co., Ann Arbor, MI, USA) using a previously described method validated for tuatara (Anderson *et al*., 2014). Briefly, CORT was extracted from plasma samples with redistilled dichloromethane, and each sample was assayed in duplicate. For each extraction, a subset of tritiated CORT samples were analysed to measure extraction recovery. Extraction recovery was 106 ± 8% (mean ± SD) with an overall coefficient of variation of 7%. Intra-assay and interassay coefficients of variation were 9.9 and 14.2%, respectively.

### Statistical analyses

Data analyses were carried out using R v3.2.0 statistical software (R Development Core, 2013) and Prism 6 (Graphpad Software Inc., La Jolla, CA, USA). All data were checked for assumptions of normality and were transformed if necessary. Linear mixed effects regression (LMER) models were fitted using the ‘lme4’ package (Bates, 2013) in R to analyse the following: (i) the acute CORT response during the initial translocation process; and (ii) the long-term dynamics of CORT secretion in translocated populations. Models were constructed through forward/backward stepwise regression procedures (Field *et al*., 2012). In all LMER models, a random effect of tuatara identity was included to account for repeat sampling of individuals. The ‘lmerTest’ package (Kuznetsova, 2013) was used to compute *P*-values for coefficients in final models, and significance was assumed at *P* < 0.05.

Sex (M, F) and linear covariates of body temperature (*T*_b_) and body condition score (log tail-corrected mass/log snout–vent length) were not significant predictors of CORT secretion in this study (LMER, *P* > 0.05) and were therefore not included in final models. Furthermore, the location of release site (within translocated populations) did not have a significant effect on CORT secretion in either translocation study A (*P* = 0.775) or B (*P* = 0.656); therefore, individuals from separate release sites within translocated populations were pooled for further analyses.

In analysis 1 (short-term monitoring), log-transformed CORT was the response variable, and sample (baseline, 18 h hold, 42 h hold, 42 h hold + processing + transfer or 66 h hold + processing + transfer) was the input variable. In analysis 2 (long-term monitoring), we first examined whether CORT secretion varied by release site within translocated populations, with log-transformed CORT the response variable and input variables of hour (0 or 3 h), site (site 1 or site 2) and an interaction term of hour × site. Models were fitted to data from Motuihe Island and Sanctuary Mountain Maungatautari, because these translocated populations had two separate release locations. Then, we compared CORT secretion between translocated and source (control) populations, with log-transformed CORT the response variable and input variables of hour (0 or 3 h), sample (source pre-, source post-, translocated pre- or translocated post-) and an interaction term of hour × sample. Lastly, we compared body condition pre- and post-­translocation, with body condition score (log mass/log snout–vent length) the response variable and sample (source pre-, source post-, translocated pre- or translocated post-) the input variable. Models were fitted to data from translocation A and B.

## Results

### Short-term monitoring: the acute ­corticosterone response during different stages of the translocation process

**Figure 3:**
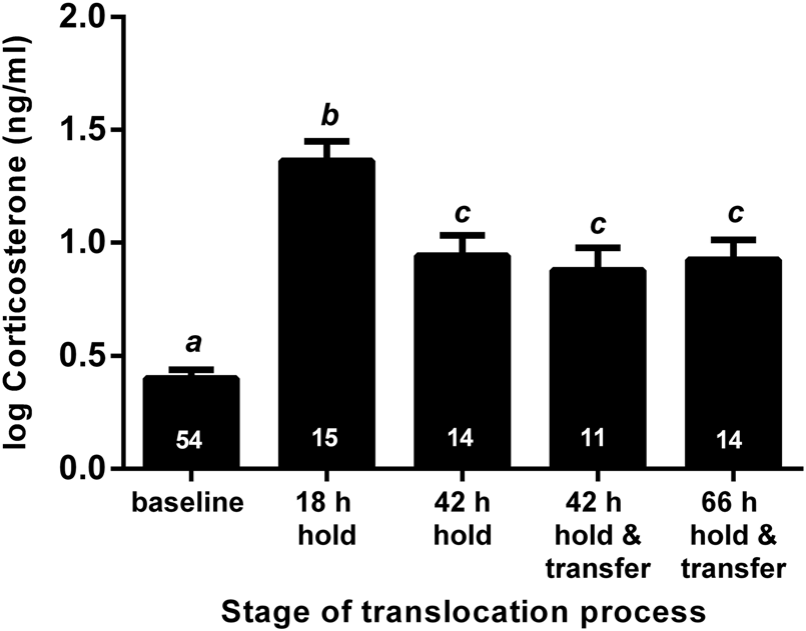
Short-term monitoring. The corticosterone response (in nanograms per millilitre) of tuatara (*Sphenodon punctatus*) at different stages of the translocation process during translocation A to Motuihe Island from Lady Alice Island in March 2012. Sample size (*n*) is indicated by the number at the base of each bar. Bars that share identical letters are not significantly different (*P* > 0.05).

An acute CORT response (indicated by a significant increase from baseline CORT) was observed in all stages of the ­translocation process of tuatara from Lady Alice Island to Motuihe Island (Table 1 and Fig. 3). The acute CORT response peaked at 18 h hold and successively decreased (though remaining significantly higher than baseline CORT) at 42 h hold (LMER, *t* = −4.495, *P* < 0.001), 42 h hold + processing + transfer (LMER, *t* = −3.899, *P* < 0.001) and 66 h hold + processing + transfer (LMER, *t* = −4.118, *P* < 0.001; Fig. 3). Contrary to our prediction, cumulative procedures of processing + transfer did not amplify the acute CORT response, because individuals held for 42 h (without processing + transfer) showed a similar acute CORT response to individuals held for 42 h + processing + transfer (LMER, *t* = 0.305, *P* = 0.761) and to individuals held for 66 h + processing + air transfer (LMER, *t* = 0.371, *P* = 0.712). Corticosterone concentrations in animals experiencing the latter three treatments were significantly lower than in individuals held for 18 h only (Table 1 and Fig. 3).

**Table 1:** Results from a linear mixed-effects regression model examining corticosterone secretion (in nanograms per millilitre) during different stages of the translocation process

Stages of translocation process	Estimate	SE	*t*-value	*P*-value
(Intercept)	0.400	0.038	10.39	<0.001
18 h hold	0.956	0.077	12.42	<0.001
42 h hold	0.496	0.079	6.25	<0.001
42 h hold + process + transfer	0.530	0.088	6.01	<0.001
66 h hold + process + transfer	0.535	0.079	6.74	<0.001

Coefficient estimates (positive or negative) are shown and indicate the ­direction of the linear regression from the intercept (baseline corticosterone 0 h). Standard errors (SE), *t*-values and *P*-values are shown.

### Long-term monitoring: dynamics of ­corticosterone secretion (baseline CORT and the CORT response) in translocated ­populations

**Figure 4:**
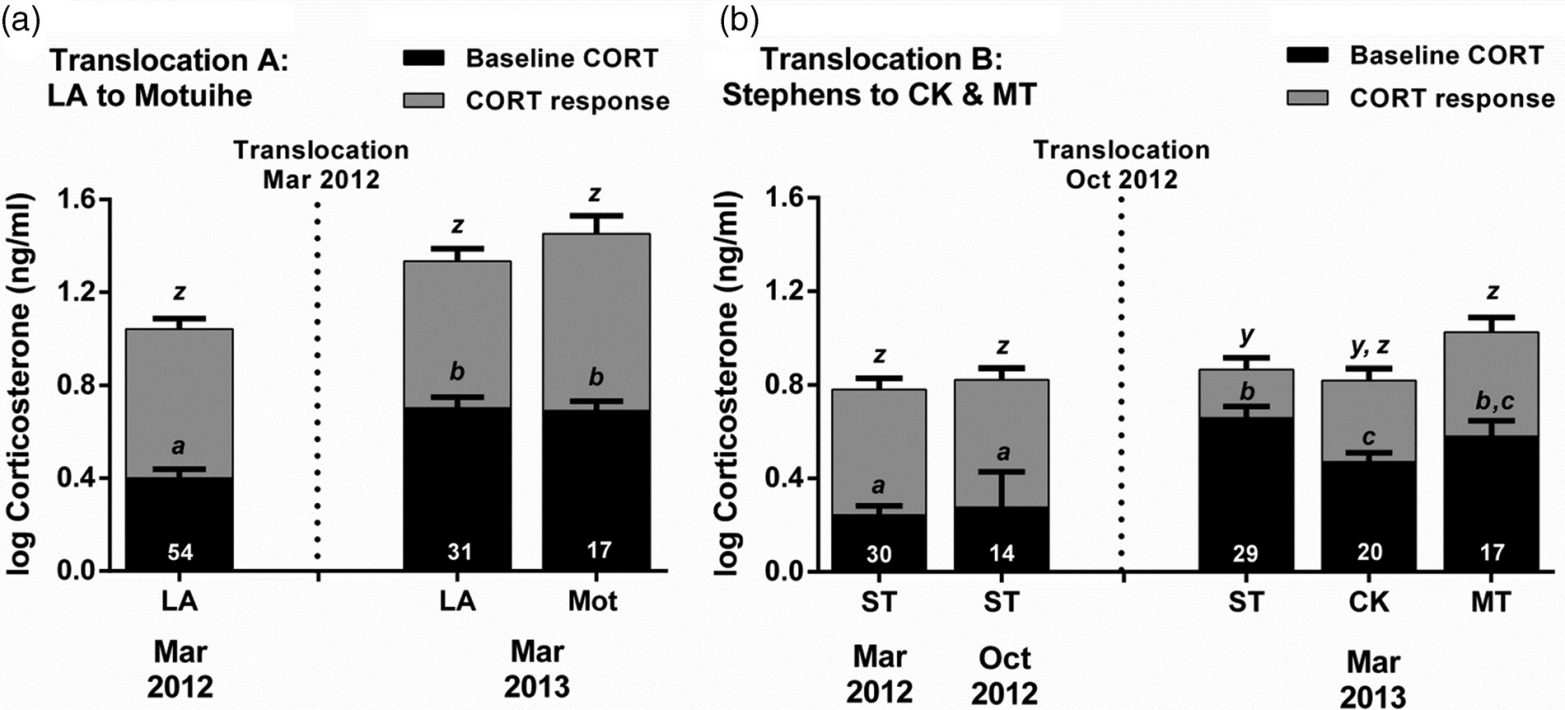
Long-term monitoring. Dynamics of corticosterone (CORT) secretion (shown as means + SEM) in populations of tuatara (*S. punctatus*) translocated to: (**a**) Motuihe Island from Lady Alice Island (LA); and (**b**) Cape Kidnappers Sanctuary (CK) and Sanctuary Mountain Maungatautari (MT) from Stephens Island (ST). Sample size (*n*) is indicated by the number at the base of each bar and represents a paired sampled of baseline CORT (black bars) and the CORT response (3 h minus 0 h; grey bars) taken from all individuals. Bars that share identical letters are not significantly different (*P* > 0.05).

In translocation A, CORT secretion was similar between translocated (Mot) and source (LA) populations at 12 months post-translocation (baseline CORT, LMER, *t* = −0.163, *P* = 0.871; CORT response, LMER, *t* = 1.136, *P* = 0.259; Fig. 4a). In both populations, baseline CORT was significantly higher at 12 months post-translocation (March 2013) compared with pre-translocation (March 2012); however, the CORT response was similar pre- and post-translocation (Table 2a and Fig. 4a).

**Table 2:** Results from linear mixed effects regression models examining dynamics of corticosterone secretion (in nanograms per millilitre) pre- and post-translocation to Motuihe Island from Lady Alice Island (source population; a) and to Cape Kidnappers and Sanctuary Mountain Maungatautari from Stephens Island (source population; b)

Long-term corticosterone dynamics post-translocation	Estimate	SE	*t*-value	*P*-value
(a) Translocation A: LA to Motuihe				
(Intercept)	0.401	0.040	9.97	<0.001
Hour	0.641	0.050	12.71	<0.001***
Post-translocation (source LA)	0.306	0.066	4.60	<0.001***
Post-translocation (translocated Motuihe)	0.291	0.082	3.55	<0.001***
Hour × post-translocation (source LA)	−0.013	0.083	−0.15	0.875
Hour × post-translocation (translocated Motuihe)	0.115	0.104	1.10	0.271
(b) Translocation B: ST to CK and MT				
(Intercept)	0.244	0.050	4.89	<0.001
Hour	0.536	0.070	7.58	<0.001***
Pre-translocation (October source ST)	0.032	0.088	0.36	0.715
Post-translocation (March source ST)	0.414	0.071	5.81	<0.001***
Post-translocation (March translocated CK)	0.227	0.079	2.88	0.004**
Post-translocation (March translocated MT)	0.337	0.083	4.05	<0.001***
Hour × pre-translocation (October source ST)	0.008	0.125	0.06	0.945
Hour × post-translocation (March source ST)	−0.329	0.100	−3.26	0.001**
Hour × post-translocation (March translocated CK)	−0.188	0.111	−1.68	0.093
Hour × post-translocation (March translocated MT)	−0.093	0.117	−0.79	0.427

Coefficient estimates (positive or negative) are shown and indicate the direction of the linear regression from the intercept (baseline corticosterone 0 h). Standard error (SE), *t*-values and *P*-values are shown. Abbreviations: CK, Cape Kidnappers; LA, Lady Alice Island; MT, Sanctuary Mountain Maungatautari; ST, Stephens Island.

In translocation B, CORT secretion varied between translocated (CK and MT) and source (ST) populations at 6 months post-translocation. Baseline CORT was significantly lower in one translocated population (CK; LMER, *t* = −2.345, *P* = 0.020), but was similar in the other translocated population (MT; LMER, *t* = −0.925, *P* = 0.356) compared with the source (ST) population (Fig. 4b). The CORT response was similar in one translocated population (CK; LMER, *t* = −1.247, *P* = 0.213), but was significantly higher in the other translocated population (MT; LMER, *t* = 1.991, *P* = 0.048) compared with the source (ST) population (Fig. 4b). Corticosterone secretion (both baseline CORT and the CORT response) was similar between the two translocated populations (CK and MT; baseline CORT, LMER, *t* = 1.210, *P* = 0.227; and CORT response, LMER, *t* = 0.745, *P* = 0.457; Fig. 4b). Corticosterone secretion in the source (ST) population was similar between both pre-translocation samples (March 2012 vs. October 2012; Table 2b and Fig. 4b). In all populations, baseline CORT was significantly higher at 6 months post-translocation (March 2013) compared with both pre-translocation samples (March 2012 and October 2012; Table 2b and Fig. 4b). The CORT response was similar pre- and post-translocation in the two translocated populations (CK and MT), but was significantly lower post-translocation in the source (ST) population (Table 2b and Fig. 4b).

### Body condition

**Figure 5:**
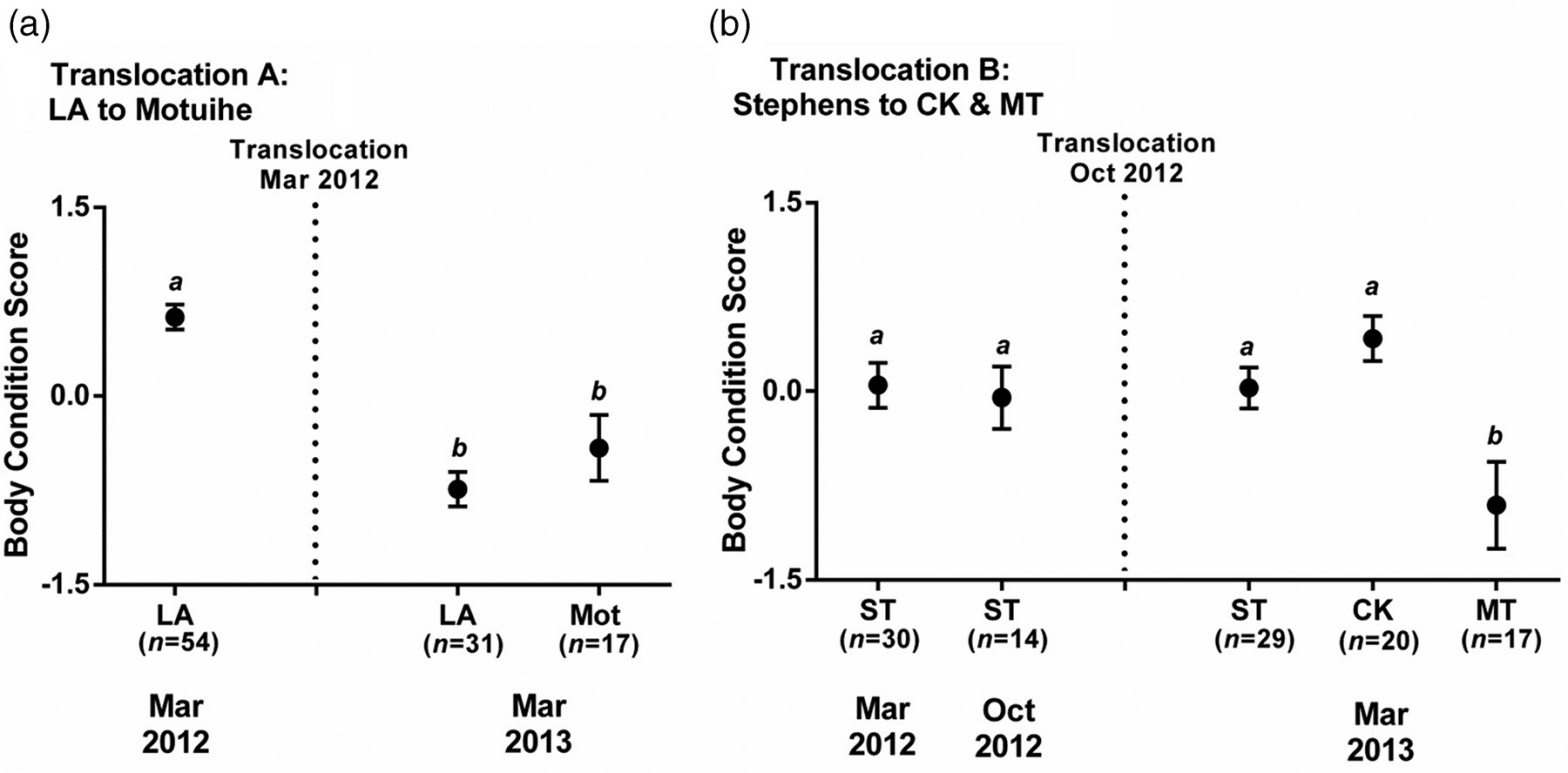
Body condition scores (log mass/log snout–vent length) in populations of tuatara (*S. punctatus*) translocated to: (**a**) Motuihe Island from Lady Alice Island (LA); and (**b**) Cape Kidnappers Sanctuary (CK) and Sanctuary Mountain Maungatautari (MT) from Stephens Island (ST). Data points (means ± SEM) that share identical letters are not significantly different (*P* > 0.05).

In translocation A, body condition was similar between translocated (Mot) and source (LA) populations at 12 months post-translocation (LMER, *t* = 1.342, *P* = 0.183; Fig. 5a). In both populations, body condition was significantly lower at 12 months post-translocation (March 2013) compared with pre-translocation (March 2012; Mot, LMER, *t* = −4.632, *P* < 0.001; and LA, LMER, *t* = −7.514, *P* < 0.001; Fig. 5a).

In translocation B, body condition at 6 months post-translocation was similar in one translocated population (CK; LMER, *t* = −1.351, *P* = 0.179) but was significantly lower in the other translocated population (MT; LMER, *t* = −3.058, *P* = 0.003) compared with the source (ST) population (Fig. 5b). Furthermore, body condition in the translocated populations (CK and MT) varied, because body condition was significantly lower in the MT population compared with the CK population (LMER, *t* = −4.022, *P* < 0.001; Fig. 5b). Body condition was similar post-translocation (March 2013) compared with pre-translocation (March 2012 and October 2012) in the translocated (CK) and source (ST) populations, but was significantly lower in the translocated (MT) population (CK, LMER, *t* = 1.261, *P* = 0.210; and MT, LMER, *t* = −3.083, *P* = 0.003; ST: LMER, *t* = −0.077, *P* = 0.938, Fig. 5b).

## Discussion

Here, for the first time, we examined CORT secretion throughout the entire translocation process in a rare reptile (the tuatara, *S. punctatus*). Our findings were as follows: (i) plasma CORT concentrations remain elevated throughout the initial translocation process (short-term monitoring between 18 and 66 h) but are not amplified by cumulative stressors; and (ii) the long-term dynamics of CORT secretion are similar in translocated and source populations. Taken together, our results show that tuatara are generally resilient to cumulative acute stressors and to chronic translocation stress.

### Cumulative stressors during translocation do not affect the acute corticosterone response in tuatara

To our knowledge, this is the first study to quantify the effect of cumulative stressors (routinely experienced in a translocation) on the acute CORT response in a reptile. We expected to see an effect of additive stressors on the acute CORT response, but this was not the case. The CORT response peaked at 18 h of holding/captivity restraint, and additional processing procedures of measurements, microchip insertion and air transfer did not increase CORT secretion further, suggesting resistance to cumulative stressors in this species. Some species show diel variation of CORT secretion (Breuner *et al*., 1999; Jones and Bell, 2004), which can confound interpretation of results if samples are not taken at 24 h intervals; however, no evidence of a diel cycle has been found in the tuatara (Tyrrell and Cree, 1998). Nevertheless, a significant CORT response was observed throughout all stages of the translocation process, and at no point did CORT returned to baseline concentrations. This observation is consistent with results from our previous study examining the acute CORT response to capture restraint in tuatara, in which a return to baseline CORT concentrations was not observed over 24 h (Anderson *et al*., 2014). Therefore, we recommend that animal disturbance, holding time, transport duration and post-translocation disturbance be minimized in tuatara to mitigate potentially harmful effects of sustained CORT secretion in individuals directly following translocation.

In the present study, we did not examine patterns of CORT secretion in the immediate weeks following translocation (our first follow-up sampling occurred at 6 months post-translocation). Consequently, we are lacking information on the speed of recovery to baseline CORT secretion levels. Langkilde and Shine (2006) found that CORT secretion in male and female lizards (*Eulamprus heatwolei*) subjected to microchip ­implantation remained elevated at 14 days (post-treatment) and ­subsequently increased in response to additional stressors at that time. Likewise, tortoises (*Testudo hermanni*) that experienced handling plus ground transport had increased baseline CORT at 4 weeks (post-stressor), compared with a control group that experienced handling only (Fazio *et al*., 2014). These studies have shown that short-term CORT secretion dynamics are significantly altered by processes experienced during a translocation; therefore, obtaining supplementary information on short-term patterns (within 4 weeks post-translocation) of CORT secretion in tuatara would shed light on the presence of a sustained CORT response and speed of recovery/negative feedback dynamics following translocation.

### Long-term dynamics of corticosterone secretion in tuatara are not altered by ­translocation

We found that translocation of tuatara did not consistently result in altered CORT secretion relative to control animals (source populations) at 6 or 12 months following translocation (summarized in Table 3). Our results accord with recent studies of translocated reptiles in which CORT secretion was not altered post-translocation. For example, Drake *et al*. (2012) found that baseline CORT in desert tortoises (*Gopherus agassizii*) was similar between translocated and control groups at both 1 and 2 years post-translocation, and both Holding *et al*. (2014) and Heiken (2013) found that baseline CORT and the CORT response in translocated northern pacific rattlesnakes (*Crotalus oreganus*) were not altered post-translocation, compared with controls. In contrast, Gerber *et al*. (2004) found that baseline CORT in translocated Turks and Caicos iguanas (*Cyclura carinata*) remained significantly higher than controls at 1, 5 and 12 months following translocation; however, body condition improved and successful reproduction occurred in translocated animals. Although studies are few, our results add to the general reported trend of resilience to translocation and/or translocation stress in reptiles. In contrast, several studies in mammals and birds have reported significant long-term effects of ­translocation on CORT secretion (Franceschini *et al*., 2008; Dickens *et al*., 2009; Zidon *et al*., 2009; Gelling *et al*., 2012; Jachowski *et al*., 2013). However, this observation is not consistent, because other studies have reported no long-term effect (Hartup *et al*., 2005; Adams *et al*., 2013; Bosson *et al*., 2013; Ji *et al*., 2013), suggesting that adaptation to new environments (indicated by long-term CORT secretion) is species specific or context dependent (e.g. might be due to time of year, weather conditions or hard vs. soft release).

**Table 3:** Summary of long-term dynamics of baseline CORT, the 3 h CORT response and body condition at 12 months post-translocation in source (Lady Alice Island or Stephens Island) and translocated populations (Motuihe Island, Cape Kidnappers or Sanctuary Mountain)

Population	Baseline CORT	CORT response	Body condition
Lady Alice Island	↑ ***	No change	↓ ***
Motuihe Island	↑ ***	No change	↓ ***
Stephens Island	↑ ***	↓ **	No change
Cape Kidnappers	↑ **	No change	No change
Sanctuary Mountain	↑ ***	No change	↓ ***

Arrows indicate direction of change and asterisks denote level of significance (**P < 0.01 and ***P < 0.001). Abbreviation: CORT, corticosterone.

Unexpectedly, through long-term monitoring in this study, we observed a significant annual increase in baseline CORT among all source and translocated populations (Table 3 and Fig. 4), probably indicating a ubiquitous environmental effect. The CORT response was unaltered in all populations, with the exception of the Stephens Island source population, where the CORT response was reduced at 12 months post-­translocation (Table 3). Body condition declined in the Lady Alice source population and the Motuihe Island and Cape Kidnappers translocated populations. Moreover, these results highlight the importance of collecting information simultaneously from source populations (as a control), because without this our results of increased baseline CORT in all translocated populations and reduced body condition in two out of three translocated populations could have been erroneously interpreted as an indication of chronic stress.

It is probable that we detected an unplanned/unexpected effect of drought on baseline CORT secretion in tuatara. In 2012–2013, New Zealand experienced its worst drought in 40 years, with the North Island affected more severely (Porteous and Mullan, 2013). Lance *et al*. (2010) observed increased plasma CORT in alligators (*Alligator mississippiensis*) experiencing a severe drought, and recovery of CORT concentrations (to within normal limits) was observed after substantial rainfall. Although dehydration stress was not directly measured (by way of CORT secretion), Davis and DeNardo (2009) found that water supplementation in a long-lived desert lizard (the Gila monster, *Heloderma suspectum*) led to greater hydration, tail-fat reserves and surface activity. In a recent experimental study, Dupoue *et al*. (2014) examined CORT secretion in water-deprived snakes (*Antaresia childreni*) and found that the CORT response, but not baseline CORT, was significantly higher in dehydrated snakes, and the loss of body mass was two to four times greater, compared with controls. The authors suggest that baseline CORT in snakes may respond only to a more severe degree of dehydration and that reduced locomotion (to reduce levels of dehydration) may explain the amplified CORT response in water-deprived snakes (Dupoue *et al*., 2014).

Reptiles, including tuatara, can moderate water loss through behavioural adaptations, such as limiting movement/locomotion and retreating to (or not emerging from) burrows, caves, fallen logs or undersides of rocks, where humidity is higher (Wilson *et al*., 2001; Bonnet and Brischoux, 2008; Davis and DeNardo, 2009; Cree, 2014). Dunlap (1995) found that lizards (*Sceloporus occidentalis*) that were more active (compared with less active) during a drought experienced greater changes in physiological measures (e.g. CORT, weight loss, haematocrit and osmolality). Moreover, Dunlap (1995) suggested that individual variation in behavioural responses of reptiles (e.g. remaining active during drought) can lead to biased analysis of stress in natural populations. Burrowing in tuatara reduces water loss by up to three times the rate experienced when emerged (Cree, 2014). Thus, it is possible that the increased baseline CORT observed in our study is influenced by sampling bias (capturing active individuals out of burrows rather than inactive individuals remaining in burrows).

Contrary to our prediction, an amplified CORT response (post-translocation) was not observed in translocated populations experiencing a shift to warmer climates/lower latitudes, specifically the Cape Kidnappers and Sanctuary Mountain translocated populations. In previous studies, we observed an amplified CORT response in tuatara at higher temperatures (L. Anderson, N. Nelson and A. Cree, unpublished data) and at lower latitudes (L. Anderson, N. Nelson, D. Towns and A. Cree, unpublished data). The Stephens Island (40° 40′ S) source population showed a reduced CORT response (from pre-translocation to post-translocation), which was not observed in the Cape Kidnappers (39° 64′ S) and Sanctuary Mountain (38° 30′ S) translocated populations (in which the CORT response was unaltered). Likewise, individuals translocated to Motuihe Island (35° 58′ S) from Lady Alice Island (35° 53′ S) did not show an altered CORT response. It is possible that the individuals translocated from Stephens Island (to Cape Kidnappers and Sanctuary Mountain) would have shown a dampened CORT response (at 12 months) if they remained on Stephens Island or were translocated to equal or higher latitudes. Examining CORT secretion in tuatara populations translocated to equal/higher latitudes [e.g. to Orokonui Ecosanctuary (45° 77′ S) from Stephens Island; Cree, 2014], might clarify the effects of latitudinal/climate change on the CORT response.

Although body condition did not statistically influence CORT secretion in our study, the sustained body condition in the Cape Kidnappers translocated population and in the Stephens Island source population suggests better hydration at these sites in the midst of a drought. Porteous and Mullan (2013) report that New Zealand's South Island (close to where Stephens Island is located) was not affected as severely as the North Island, and better hydration in the Cape Kidnappers population was probably achieved through provision of supplementary water sources (L. Anderson, personal observation). Reduced body condition has been observed in dehydrated/water-restricted reptiles, including snakes (Dupoue *et al*., 2014; Lillywhite *et al*., 2014), lizards (Summers and Norman, 1988; Dunlap, 1995; Davis and DeNardo, 2009, 2010), alligators (Lance *et al*., 2010) and turtles (Ray *et al*., 2004, 2008; van de Merwe *et al*., 2013). Clearly, information on relationships among water availability/dehydration, body condition, stress and CORT secretion is lacking and should be considered in light of imminent ­climate change.

## Funding

This work was supported by Victoria University of Wellington, the Allan Wilson Centre for Molecular Ecology and Evolution, the Centre for Biodiversity and Restoration Ecology and the San Diego Zoo.
